# AN OLDER ADULT FRACTURE PROGRAM EMPLOYING AGE-FRIENDLY CARE CAN BE IMPLEMENTED AT YOUR HOSPITAL AND IMPROVE OUTCOMES

**DOI:** 10.1093/geroni/igac059.2725

**Published:** 2022-12-20

**Authors:** Kathleen Breda, Michelle Keller, Hiroshi Gotanda, Karma McKelvey, Carol Lin, Sonja Rosen

**Affiliations:** Cedars-Sinai Medical Center, Los Angeles, California, United States; Cedars-Sinai Medical Center, Los Angeles, California, United States; Cedars-Sinai Medical Center, Los Angeles, California, United States; Cedars-Sinai Medical Center, Los Angeles, California, United States; Cedars-Sinai Medical Center, Los Angeles, California, United States; Cedars-Sinai Medical Center, Los Angeles, California, United States

## Abstract

Older adult fractures are expected to increase dramatically over the next several decades, with hip fractures reaching an estimated 6.26 million worldwide by 2050. A combined approach to the care of older adult fracture patients was first reported in the early 1990s, emphasizing geriatric and orthopedic co-management. Early studies showed significant improvements in complications, function, and discharge rates to nursing homes. Subsequent studies demonstrated shorter time-to-surgery, lengths of stay (LOS), lower readmission rates, reduced in-hospital mortality, and lower cost of care. The studies are from large academic centers whose physicians are employed faculty or have dedicated geriatric units. Less studied is whether similar models could be implemented in community health systems/mixed practice settings like Cedars-Sinai. Cedars-Sinai’s older adult fracture program integrates inpatient and outpatient care and uses best practices of the 4Ms Framework of an Age-Friendly Health System, including What Matters Most, Mentation, Medications, and Mobility. The program was designed through the consensus of interprofessional champions and empirical evidence. Results showed enrolled patients with all fracture types had significantly lower LOS (marginal effect [ME]: -2.12, 95%CI: -2.61, -1.63), LOS index (ME: -0.33, 95%CI: -0.42, -0.25), and total direct costs (ME: -$5316.4, 95%CI: -6806.31, -3826.5). There was no significant difference in time-to-surgery. Of the 746 enrolled patients, 170 (23%) had a post-discharge visit with a participating geriatrician within 6 months. Findings indicate a systematic approach to improving care for older adults with fractures improves inpatient outcomes in a mixed practice setting. Future studies will examine the effectiveness of Cedar-Sinai’s outpatient program.

